# University of Pennsylvania

**Published:** 1880-03

**Authors:** 


University of Pennsylvania, Department of Dentistry.—Our friend Prof. Chas. J. Essig, D. D. S., M. D., Secretary, sends us the following information: At a public commencement held Monday, March 15, 1880, at the American Academy of Music, the Degree of Doctor of Dental Surgery was conferred by Charles J. Stillé, LL.D., Provost, upon twenty gentlemen; after which an address was delivered by Harrison Allen, M. D., Professor of Physiology. Prizes—Distinguished Merit and Honors, E. C. Weston, Philadelphia—one prize for best essay, another for best specimen of plate work, and his degree with honor. John N. P. Newton, L. D. S., England, Thesis of Distinguished Merit, and his degree with honor. W. G. Matthews, Philadelphia, prize for best specimen of platinum work and degree with honor. John A. Klump, Pa., Thesis of Distinguished Merit. Homan Portuondo, Cuba, Thesis of Distinguished Merit. Number of matriculates, 77.

Reprint pamphlets received. On the "Physiological Action of the Muscles Concerned in the Movements of the Lower Jaw." "The Larynx, the Source of the Vowel Sounds." "Hard Rubber Appliance for Congenital Cleft Palate." All three by and from Thomas Bryan Gunning, M. D., of New York. "The Distribution of Living Matter in Human Dentine, Cement and Enamel." "Necrosis." "On Secondary Dentine." "On Pericementum and Pericementitis." The four by and from C. F. W. Bödecker, D. D. S., M. D. S., of New York. Both of these authors have treated their subjects well and scientifically.

"Le Progrès Dentaire," VII., No. 1, January, 1880. C. Ash & Sons, Paris, is our first exchange from France. We receive it with a hearty welcome.

Received Vol. I., No. 4, of "The Dental Luminary," a quarterly quarto journal, edited by Drs. J. P. and W. R. Holmes, Macon, Ga. It is handsomely printed on fine pink-tinted paper for 50 cents per annum, and filled with excellent matter. May its light brighten many offices and reflect happiness to its Ho(l)mes.

